# 

**Published:** 1934

**Authors:** 


### CONTENTS.

| | Page |
|---|---|
| The Place of Surgery in Hyperthyroidism. By Sir Thomas P. Dunhill, K.C.V.O., F.R.A.C.S. .. .. .. .. .. .. .. | 155 |
| Medical Practice in Palestine. By H. J. Orr-Ewing, M.D., F.R.C.P. | 163 |
| The Clinical Importance of the Intervertebral Discs, with Special Reference to Nuclear Prolapses. By Gilbert B. Bush, M.B., Ch.B., D.M.R.E. .. .. .. .. .. .. .. | 173 |
| Reviews of Books .. .. .. .. .. .. .. .. | 183 |
| Editorial Notes .. .. .. .. .. .. .. .. | 193 |
| Obituaries: Arthur Bernard Cridland, F.R.C.S. .. .. .. | 197 |
| Newman Neild, M.B., Ch.B., F.R.C.P. .. .. .. | 198 |
| William Wilfrid King, F.R.C.S. .. .. .. .. | 200 |
| The Medical Library of the University of Bristol .. .. | 201 |
| Publications Received .. .. .. .. .. .. .. | 203 |
| Local Medical Notes .. .. .. .. .. .. .. .. | 204 |

